# Clinical and Radiological Predictors of Biochemical Response to First-Line Treatment With Somatostatin Receptor Ligands in Acromegaly: A Real-Life Perspective

**DOI:** 10.3389/fendo.2021.677919

**Published:** 2021-05-07

**Authors:** Federica Nista, Giuliana Corica, Lara Castelletti, Keyvan Khorrami, Claudia Campana, Francesco Cocchiara, Gabriele Zoppoli, Alessandro Prior, Diego Criminelli Rossi, Gianluigi Zona, Diego Ferone, Federico Gatto

**Affiliations:** ^1^ Endocrinology Unit, Department of Internal Medicine and Center of Excellence for Biomedical Research, University of Genoa, Genoa, Italy; ^2^ Department of Radiology, Tigullio Hospital, Lavagna, Italy; ^3^ Department of Internal Medicine, University of Genoa and IRCCS Ospedale Policlinico San Martino, Genoa, Italy; ^4^ Division of Neurosurgery, Department of Neurosciences, Rehabilitation, Ophthalmology, Genetics, Maternal and Child Health, IRCCS Ospedale Policlinico San Martino, Genoa, Italy; ^5^ Endocrinology Unit, IRCCS Ospedale Policlinico San Martino, Genoa, Italy

**Keywords:** acromegaly, predictors, biochemical response, first-line therapy, somatostatin receptor ligands

## Abstract

**Background:**

First-generation somatostatin receptor ligands (fg-SRLs) represent the first-line medical treatment for acromegaly, recommended in patients with persistent disease after neurosurgery, or when surgical approach is not feasible. Despite the lack of strong recommendations from guidelines and consensus statements, data from national Registries report an increasing use of medical therapy as first-line treatment in acromegaly.

**Objective:**

We retrospectively evaluated the potential role of a large number of clinical and radiological parameters in predicting the biochemical response to 6-month treatment with fg-SRLs, in a cohort of naïve acromegaly patients referred to a single tertiary center for pituitary diseases.

**Methods:**

Univariable and multivariable logistic regression and linear regression analyses were performed. Biochemical response was defined based on IGF-1 levels, represented as both categorical (tight control, control, >50% reduction) and continuous (linear % reduction) variables.

**Results:**

Fifty-one patients (33 females, median age 57 years) were included in the study. At univariable logistic regression analysis, we found that younger age (≤ 40 years; OR 0.04, p=0.045) and higher BMI (OR 0.866, p=0.034) were associated with a lower chance of achieving >50% IGF-1 reduction. On the contrary, higher IGF-1 xULN values at diagnosis (OR 2.304, p=0.007) and a T2-hypointense tumor (OR 18, p=0.017) were associated with a significantly higher likelihood of achieving >50% IGF-1 reduction after SRL therapy. Of note, dichotomized age, IGF1 xULN at diagnosis, and T2-hypointense signal of the tumor were retained as significant predictors by our multivariable logistic regression model. Furthermore, investigating the presence of predictors to the linear % IGF-1 reduction, we found a negative association with younger age (≤ 40 years; β -0.533, p<0.0001), while a positive association was observed with both IGF-1 xULN levels at diagnosis (β 0.330, p=0.018) and the presence of a T2-hypointense pituitary tumor (β 0.466, p=0.019). All these variables were still significant predictors at multivariable analysis.

**Conclusions:**

Dichotomized age, IGF-1 levels at diagnosis, and tumor T2-weighted signal are reliable predictors of both >50% IGF-1 reduction and linear % IGF-1 reduction after 6 month fg-SRL treatment in naïve acromegaly patients. These parameters should be considered in the light of an individualized treatment for acromegaly patients.

## Introduction

Acromegaly is a rare, chronic, and systemic disease characterized by an excessive production and secretion of growth hormone (GH), resulting in high circulating levels of insulin-like growth factor 1 (IGF-1). In the vast majority of cases, the disease is caused by the presence of a GH-secreting pituitary tumor (1). According to recent epidemiological data, acromegaly has an estimated prevalence of 3-14 cases/100,000 people, with an annual incidence of 0.2-1.1 cases/100,000 people/year (2, 3).

Acromegaly is characterized by a broad range of clinical manifestations and several comorbidities, including metabolic impairment, cardiovascular and respiratory diseases, osteoarticular complications, thus leading to reduced quality of life and higher mortality risk compared to the general population (4, 5). All these aspects are associated with the long-term exposure to inappropriately high circulating levels of GH and IGF-1 (6). Late diagnosis is an important issue in acromegaly, since it can be delayed up to 10 years after the onset of early symptoms (1, 7, 8). Nowadays, surgical resection of the pituitary tumor performed by a skilled neurosurgeon is the first treatment choice in most patients (9, 10). As for medical therapies, first-generation somatostatin receptor ligands (fg-SRLs), octreotide (OCT) and lanreotide (LAN), still represent the first-line approach, being recommended in those patients with persistent disease after neurosurgery, or when the surgical approach is not feasible (9–11). In more detail, latest Consensus Statements and guidelines on acromegaly suggest to consider first-line treatment with SRLs in patients with contraindications to or who refuse surgery, and in those subjects considered at poor risk for good outcomes and surgical success (9, 10). Moreover, data concerning preoperative treatment with SRLs are conflicting in terms of improved surgical outcomes and biochemical control, and therefore their use in treatment-naïve acromegaly patients is still debated (11–15).

However, fg-SRL treatment has well-recognized positive effects in patients with acromegaly, both in terms of absolute and relative GH and IGF-1 reduction, achievement of biochemical control (about 30-50% of cases), as well as tumor volume reduction (11). Therefore, despite the suggestions raised from expert panels, data from national Registries report the use of medical therapy as first-line treatment in a large number of patients (from 23% up to 60% of cases, even increasing in the last decades) (16–19).

To date, a number of studies have investigated the potential role of clinical, radiological and molecular determinants able to predict the biochemical response to SRL treatment in acromegaly, although data are hardly comparable due to the high heterogeneity among the reported studies (e.g. prospective vs. retrospective design, various definitions of biochemical control, first-line therapy vs. adjuvant treatment) (20–24). The main clinical predictors of biochemical response to fg-SRL treatment identified so far are: lower baseline IGF-1 levels and older age at diagnosis (11, 21, 22). As for the radiological features, a T2-weighted hypointense signal of the pituitary lesion has been associated with a better response to SRL therapy in acromegaly patients (25–29).

However, few studies have focused on the predictors to first-line treatment with SRLs in naïve acromegaly patients. To our opinion, this is of particular interest since, in this peculiar setting, a number of useful molecular predictors identified in previous translational studies, such as the expression of somatostatin receptor subtype 2 (SST_2_) and E-cadherin on resected tumors (30–33), as well as information about cell proliferation markers (e.g. %Ki67 labeling) (33, 34), cannot be considered. Furthermore, most studies investigating the response to SRL first-line treatment have been carried out in the setting of preoperative SRL therapy, therefore focusing on the impact of medical treatment on both short- and long-term surgical outcome, as well as surgical complications (12–15).

Overall, the identification of robust clinical and radiological predictors of the biochemical response to first-line SRL treatment in acromegaly, feasible in all referral center for pituitary diseases, would benefit the debate concerning the management of those patients in which the decision for a direct surgical approach is not clean-cut.

Therefore, the aim of the present study was to retrospectively evaluate the potential role of a large number of clinical and radiological parameters in predicting the biochemical response to 6-month treatment with fg-SRLs in a cohort of naïve acromegaly patients referred to a single tertiary center for pituitary diseases. For this purpose, selected variables were tested in both univariable and multivariable regression models. The identification of patient characteristics easily available in all pituitary referral centers as valuable predictors of treatment response represented the ultimate goal of the study.

## Patients and Methods

### Patients

Fifty-one acromegaly patients treated with fg-SRLs as first-line therapy, followed-up at the Endocrinology Unit of the IRCCS Ospedale Policlinico San Martino (Genoa, Italy), were included in the study. Diagnosis of acromegaly was made based on clinical features, biochemical evidence of GH hypersecretion (lack of suppression of GH to <1 µg/L after a 2-hour oral glucose tolerance test (OGTT), in patients without overt diabetes mellitus), IGF-1 levels above the age-adjusted upper limit of normality range (>1 xULN), and the presence of a pituitary tumor at magnetic resonance imaging (MRI). About 20% of cases were referred to our center just after diagnosis, therefore the complete results of diagnostic OGTT were not available in 19 patients.

Inclusion criteria were: i) available baseline clinical characteristics in patients’ charts (sex, age, body mass index (BMI), presence of comorbidities likely related to acromegaly, disease duration); ii) biochemical data evaluation (GH, IGF-1 levels and remaining anterior pituitary function); iii) treatment with fg-SRLs as first-line treatment for at least 6 months.

Exclusion criteria were: i) previous treatments (surgery, other medical therapies for acromegaly, radiotherapy); ii) concomitant medical therapy with the GH receptor antagonist pegvisomant and/or with the dopamine agonist cabergoline; iii) changes in the treatment schedule during the 6-month observation period.

Disease duration, and the related diagnostic delay, were assessed by the comparison of patients’ photographs and by patients’ interviews (e.g. onset of acral enlargement) (35). Complete biochemical assessment of the anterior pituitary function allowed to identify the presence of hypopituitarism or concomitant hypersecretion of other pituitary hormones.

A number of pathological conditions known to be associated with acromegaly have been recorded in all patients included in the study. In detail, we evaluated the presence of type 2 diabetes mellitus (T2DM), hypertension, cardiomyopathy, goiter, colonic polyps, carpal tunnel syndrome, obstructive sleep apnea syndrome, and impaired bone density.

General information about tumor size at diagnosis (macro- vs. microadenoma) were available in all patients. However, a detailed evaluation of MRI images at diagnosis, performed by a single neuroradiologist with large experience on pituitary imaging, was performed in 28 patients. In this subgroup, the following parameters were evaluated: maximum tumor diameter, tumor volume (estimated by the ellipsoid equation) (36), tumor invasiveness (by use of Knosp score), and T2-weighted signal intensity characteristics. In order to define the T2-weighted signal intensity of the pituitary tumors, we used the method proposed by Potorac and colleagues (37).

Visual field examination, available for all patients, was performed with a Humphrey field analyzer HFA II.

Furthermore, 22 patients underwent an acute octreotide tolerance test, as previously described (38). The test was first performed as routine clinical evaluation in our center, while in the most recent years, following the controversies raised about its real impact on patient management (39–42), this procedure was discontinued. However, available data were included in the present analysis in order to investigate whether, in our cohort, the test results could have any predictive value on patients’ response (alone or in combination with other parameters).

The study was conducted in line with the recommendations of the declaration of Helsinki and all the patients gave written informed consent to use the available data for research purpose.

### Biochemical Response to SRLs Treatment

Biochemical response to 6-month treatment with fg-SRLs was defined as: 1) tight biochemical control (last IGF-1 value ≤1 xULN); 2) biochemical control (last IGF-1 value ≤1.2 xULN); 3) reduction of IGF-1 >50% compared to the baseline value. All the above mentioned criteria are widely reported in the literature (23, 43). Furthermore, the percentage IGF-1 reduction between baseline and 6-month values (% IGF-1 reduction) was evaluated as an additional measure of fg-SRL efficacy (21). Therefore, in our prediction models, biochemical response was represented with both categorical (tight control, control, >50% IGF-1 reduction) and continuous variables (% IGF-1 reduction).

### GH and IGF-1 Assays

All samples included in the analysis were assessed in the same laboratory (Medicine Laboratory, IRCCS Ospedale Policlinico San Martino, Genova, Italy), using the same assay for both GH and IGF-1 measurements. GH levels were determined using a two-site chemiluminescent immunometric assay (Immulite 2000, Siemens Healthcare Diagnostics Products), calibrated to the WHO 98/574 International Standard (IS). The lower detection limit is 0.05 µg/L, while analytical sensitivity is 0.01 µg/L. The intra-assay and inter-assay coefficients of variation (CVs) are 2.9-4.6% and 4.2-6.6%, respectively.

IGF-1 values were evaluated with a chemiluminescent immunometric assay (Immulite 2000, Siemens Healthcare Diagnostics Products), calibrated to the WHO 87/518 IS. The assay has a detection range of 20-1600 μg/L, and an analytical sensitivity of 20 μg/L. The intra-assay and inter-assay CVs are 2.3-3.9% and 3.7-8.1%, respectively.

### Candidate Predictors

Variables that were considered as potential predictors of biochemical response and % IGF-1 reduction were selected based on available data at diagnosis, previous studies, and biological plausibilities (23, 24, 44). In more detail, in all subjects we evaluated the impact of: age [as continuous variable, and stratified into younger (≤40 years) and older (>40 years) patients], sex, BMI, diagnostic delay, GH and IGF-1 values, anterior pituitary function (presence of hyperprolactinemia and/or hypopituitarism), diabetes insipidus, tumor size (micro- vs. macroadenoma), fasting plasma glucose, T2DM and other disease-related comorbidities (see *Patients and Methods* section), visual field defects, the drug used (octreotide LAR vs. lanreotide Autogel) and the related dose.

Other relevant information potentially useful to predict fg-SRL response in our cohort were available in a subset of patients. Although considering this limitation, the following data were also included in the analysis: maximum tumor diameter, tumor volume, invasiveness (Knosp grade), T2-weighted signal intensity, nadir GH levels and the percentage GH reduction after both OGTT (performed at diagnosis) and acute octreotide tolerance test (performed before treatment start).

### Statistical Analysis

The data were analyzed using SPSS software version 25.0 for Windows (SPSS, Chicago, IL), or the R software, when appropriate. Graphs and figures were drawn by use of GraphPad Prism version 5.02 (GraphPad Software, San Diego, CA). Quantitative data are presented as median and interquartile range (IQR). The Kolmogorov-Smirnov test was used to check the normality of the distribution of the continuous variables. Between-group comparisons were analyzed by the Mann-Whitney U test or the Kruskal-Wallis test for continuous variables. Chi-square and Fischer’s exact tests were used to evaluate differences in cross-tables. Correlation coefficients were calculated using the Spearman rank order R.

The potential predictors of biochemical response after 6-month SRL therapy were identified performing summary statistics and the above described tests. Afterwards, the selected variables were included in the different prediction models. Indeed, based on the depend variables (categorical or continuous), univariable and multivariable logistic regression and linear regression analyses were performed, respectively.

To assess the point estimates and confidence intervals by univariable and multivariable analysis for variables exhibiting perfect separation with the desired outcome (e.g. dichotomized age), we employed a generalized linear model based on bias-reduction adjusted score equations (doi:10.1093/biomet/asaa052; doi:10.1093/biomet/asx046). Such analyses were conducted in R, using the package “brglm2” (doi:10.1007/s11222-019-09860-6). To avoid overfitting by multivariable analysis due to the limited number of complete observations, we employed two methods to select the features of our multivariable model, namely stepwise backward-forward selection by the Akaike information criterion (package “MASS”, doi:10.1007/978-0-387-21706-2), and feature selection by generalized linear model *via* penalized maximum likelihood with the lasso penalty (package “glmnet”, doi:10.18637/jss.v033.i01).

The receiver-operating characteristic (ROC) curve was used to assess the predictive discrimination of those parameters that were statistically significant at both univariable and multivariable analyses, with respect to biochemical response evaluated as >50% IGF-1 reduction.

## Results

### Patients Characteristics

Fifty-one acromegaly patients fulfilled the inclusion and exclusion criteria, and were therefore included in the analysis. General, clinical and biochemical patients’ characteristics, as well as pituitary tumor information available in our dataset are summarized in **Table 1**.

**Table 1:** Detailed characteristics of the acromegaly patients included in the study.

Patient characteristics	Values				
Patients (n)	51				
Sex (F/M; n, %)	F, 33 (64.7%)				
Age (median, IQR; years)	57 (45-63)				
BMI (median, IQR; Kg/m^2^)	28 (24-30)				
T2DM (n, %)	12 (23.5%)				
Pituitary deficiencies (n, %)	10 (19.6%)				
Other comorbidities (n, %)	44 (86.3%)				
Visual field defects (n, %)	26 (51.0%)				
Diagnostic delay (median, IQR; years)	7 (5-10)				
**Drugs**					
Octreotide LAR (vs. Lanreotide Autogel)	27/51 (52.9%)				
Dose (standard vs. low)	35 (68.6%)				
**Biochemical values**					
GH (median, IQR; µg/L)	12.2 (5.5-20.4)				
IGF-I (median, IQR; µg/L)	733 (538-966)				
IGF-I xULN (median, IQR; µg/L)	2.8 (2.2-4.0)				
PRL (median, IQR; µg/L)	9.4 (6.7-37.2)				
**Tumor radiological features**					
Macroadenomas (n, %)	37 (72.5%)				
Maximum diameter (n=28)* (median, IQR; mm)	14 (11-17.5)				
Tumor volume (n=28)* (median, IQR, mm^3^)	2993 (1406-7755)				
Invasiveness (n=28)*	Grade 0	Grade I	Grade II	Grade III	Grade IV
Knosp score (n, %)	15 (53.6)	10 (35.7)	2 (7.1)	1 (3.6)	0 (0)
T2-weighted MRI features (n=25)*	Hypo 11 (44)	Hypo-iso 1 (4)	Iso 5 (20)	Iso-Hyper 3 (12)	Hyper 5 (20)
Signal intensity (n, %)					
**Dynamic tests**					
OGTT at baseline (n=32)*					
GH nadir (median, IQR; µg/L)	4.8 (2.5-13.6)				
% GH reduction (median, IQR)	19% (37.4, -0.9)				
Paradoxical response (Yes; n, %)	4 (12.5%)				
Acute octreotide test (n=22)*					
GH nadir (median, IQR; µg/L)	1.1 (0.5-5.2)				
% GH reduction (median, IQR)	84.3% (73.9-94.3)				

Continuous variables are expressed as median and IQR, while categorical variables are expressed as number (n) and percentage (%). *number of patients with available information. BMI, body mass index; T2DM, diabetes mellitus type 2; octreotide LAR, octreotide long-acting release; GH, growth hormone; IGF-1, insulin-like growth factor 1; ULN, upper limit of normality range; PRL, prolactin; OGTT, oral glucose tolerance test.

At diagnosis, the median age was 57 years (IQR 45-63), with 6 patients (11.8%) ≤40 years, and 45 subjects (88.2%) >40 years. The majority of patients were females (33/51, 64.7%), of which 24 were in the post-menopausal period. No patients were undergoing oestro-progestinic treatment. The median BMI was 28 Kg/m^2^ (IQR 24-30), with only one patient classified as grade IV obesity, while 36 patients (70.5%) had values <30 Kg/m^2^.

Overall, 46 patients (90.2%) presented with at least one comorbidity likely related to acromegaly at the time of diagnosis. In detail, 12 patients (23.5%) had overt T2DM, 25 (47.1%) had hypertension and were already under medications (one subject still had elevated blood pressure), while cardiac hypertrophy was reported in 11 cases (21.6%). The presence of goiter was assessed in 31 patients (60.8%), colon polyps were detected in 11 subjects (21.6%), and carpal tunnel syndrome in 15 (29.4%). Twelve (23.5%) patients had an obstructive sleep apnea syndrome, of which 3 were undergoing continuous positive airway pressure (CPAP) treatment. The median estimated diagnostic delay was 7 years (IQR 5-10).

At baseline, the median GH levels were 12.2 µg/L (IQR 5.5-20.4), absolute IGF-1values were 733 µg/L (IQR 538-966), while IGF-1 xULN values were 2.8 xULN (IQR 2.2-4.0).

As for the remaining anterior pituitary function, the median prolactin (PRL) levels were 9.4 µg/L (IQR 6.7-37.2). No cases of panhypopituitarism were described, although 10 subjects had at least one pituitary deficit (9 patients with central hypogonadism, 1 patient with central hypothyroidism).

Twenty-six patients (51%) had visual impairments of various degrees, assessed during the visual field examination. In detail, out of the 26 patients with reported visual field impairment, 22 (85%) had mild and non-specific defects, while only 4 subjects (15%) had moderate or severe visual field impairment. Of note, 1 out of these 4 patients presented with a microadenoma (unlikely to be the primary cause of the visual impairment), while the remaining 3 patients had a macroadenoma, but they refused the neurosurgical approach as first-line treatment. Furthermore, among the 3 patients with macroadenoma and severe visual field defects, two subjects had visual impairment due to other causes than the pituitary mass (one patient with a previous retinal vein occlusion, and one patient with a proliferative diabetic retinopathy).

As concern medical therapy, 27 patients (52.9%) were treated with octreotide LAR, while 24 (47.1%) received lanreotide Autogel. We considered octreotide LAR 30 mg/4 weeks or lanreotide Autogel 120 mg/4 weeks as the standard dose of fg-SRLs, which was administered in the majority of patients (35/51, 68.6%). On the other hand, one third of patients received a low dose (defined as octreotide LAR 20 mg/4 weeks or lanreotide Autogel 90 mg/4 weeks).

As for the evaluation of the radiological parameters, most patients were diagnosed with a macroadenoma (37/51, 72.5%). Moreover, in the patients’ subgroup revised by a single skilled neuroradiologist (n=28), the median maximum diameter was 14 mm (IQR 11-17.5), tumor volume was 2993 mm^3^ (IQR 1406-7755), while only one lesion had high invasiveness, defined based on Knosp grade. When performing the qualitative analysis of the T2-weighted signal intensity, 3 tumors were classified as both hypo- and hyperintense, and were therefore excluded. Finally, out of 25 evaluable MRI, 11 patients (44%) had a T2-weighted hypointense tumor.

Considering the results of OGTT (n=32), the median GH nadir was 4.8 µg/L (IQR 2.5-13.6), with a median reduction of 19% (IQR 37.4 and -0.9), while a paradoxical rise in GH levels, defined according to the criteria proposed by Scaroni and colleagues (45), was observed in 4/32 patients (12.5%).

After performing an acute octreotide tolerance test (n=22), the median GH nadir was 1.1 µg/L (IQR 0.5-5.2), with a median GH reduction of 84.3% (IQR 73.9-94.3).

At last follow-up, tight biochemical control (IGF-1 values ≤1 xULN) and biochemical control (IGF-1 values ≤1.2 xULN) were observed in 18 (35.3%) and 23 patients (45.1%), respectively. Furthermore, 30 patients (58.8%) had a >50% IGF-1 reduction compared to baseline values. Out of the 18 patients with tight biochemical control, 16 subjects (88.9%) had a >50% IGF-1 reduction after 6-month treatment, while among the 23 patients with biochemical control, 20 subjects (87%) reached a reduction of IGF-1 >50% compared to the baseline values.

### Evaluation of Potential Predictors of Biochemical Response

#### Patients’ General and Clinical Characteristics

The median age was not significantly different between patients reaching biochemical control (all definitions) and uncontrolled ones. In line with this finding, no significant correlation was found between age and the % IGF-1 reduction after 6-month SRL treatment.

**Figure 1:**
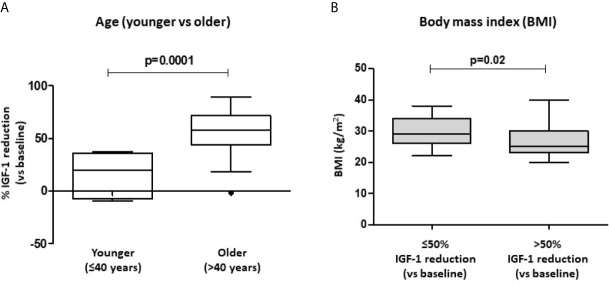
Biochemical response after first-line SRL treatment (6 months) with respect to patients’ age and BMI. Patients were stratified into younger (age ≤ 40 years) and older (>40 years) subjects. % IGF-1 reduction account for the relative IGF-1 reduction observed after 6-month therapy with fist-generation SRLs **(A)**. Significant differences in BMI values were found in patients achieving a >50% IGF-1 reduction after SRL treatment, compared to those subjects with a lower response rate **(B)**. SRLs, somatostatin receptor ligands; BMI, body mass index.

However, stratifying our patients into younger (≤40 years, n=6) and older (>40 years, n=45) subjects, we found that a lower percentage of young patients achieved a >50% IGF-1 reduction, compared to the older subjects (p=0.002). Furthermore, younger patients showed a significantly lower % IGF-1 reduction than the elderly [19.9% (IQR -7.3-36.1) vs. 58.2% (IQR 43.8-71.7); p=0.001] (**Figure 1A**).

Of note, patients that achieved a >50% IGF-1 reduction had a significantly lower BMI compared to those subjects with a poorer response to SRL treatment [25 Kg/m^2^ (IQR 23-30) vs. 29 Kg/m^2^ (IQR 26-34); p=0.020] (**Figure 1B**).

In our cohort, sex, fasting plasma glucose, T2DM, the presence of impaired pituitary function (hypopituitarism and/or diabetes insipidus), acromegaly-related comorbidities, diagnostic delay, as well as the evidence of visual field defects at diagnosis did not come out as potential predictors of response to first-line SRL treatment. Namely, these variables were not significantly different in patients reaching biochemical control compared to the uncontrolled ones.

As for treatment drugs, octreotide LAR and lanreotide Autogel were equally effective in reducing IGF-1 levels after 6-month therapy [51.6% (IQR 36.0-69.8) vs. 56.19% (IQR 25.7-71.7); p=0.940], as well as in reaching biochemical control (IGF-1 ≤1 xULN, p=0.778; IGF-1 ≤1.2 xULN, p=1.0; >50% IGF-1 reduction, p=0.777). Furthermore, drug dose did not significantly affect the evaluated outcomes.

#### Biochemical Parameters

We evaluated the role of GH, IGF-1 xULN and PRL values at diagnosis as potential predictors of biochemical response to first-line SRL therapy. Of note, the median GH and PRL values were not significantly different between patients reaching biochemical control (all definitions) and the uncontrolled ones. Furthermore, no significant correlations were found between GH levels, PRL levels and the % IGF-1 reduction after 6-month SRL treatment.

**Figure 2:**
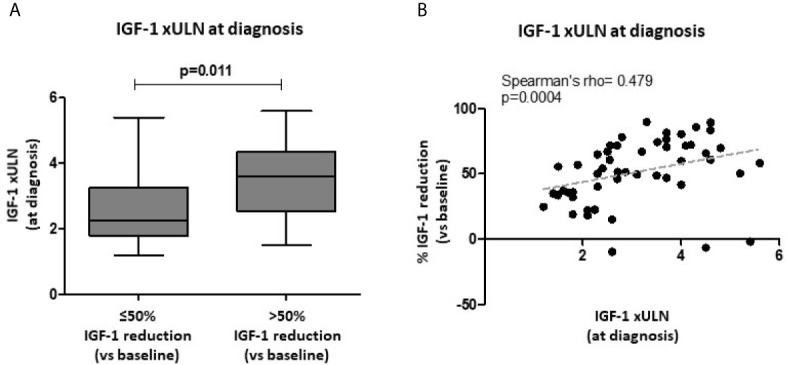
Biochemical response after first-line SRL treatment (6 months) with respect to baseline IGF-1 values. Age-adjusted IGF-1 values (xULN) were higher in patients achieving a >50% IGF-1 reduction after SRL treatment, compared to those subjects with a lower response rate **(A)**. A direct significant correlation was found between IGF-1 xULN levels at diagnosis and the % IGF-1 reduction observed after 6-month therapy with fist-generation SRLs **(B)**. SRLs, somatostatin receptor ligands; ULN, upper limit of normal.

The median IGF-1 xULN values at diagnosis were not significantly different in patients achieving biochemical control (both IGF-1 ≤1 and ≤1.2 xULN), compared to uncontrolled subjects. However, patients reaching a >50% IGF-1 reduction after treatment had higher IGF-1 xULN at baseline compared to those subjects with a lower response rate [3.6 xULN (IQR 2.6-4.4) vs. 2.3 xULN (IQR 1.8-3.3); p=0.011] (**Figure 2A**). In line with these findings, IGF-1 xULN values at diagnosis were significantly correlated with the % IGF-1 reduction observed after 6 months (Spearman’s rho=0.479, p=0.0004) (**Figure 2B**).

#### Tumor Radiological Features

Tumor characteristics such as micro- vs. macroadenoma, maximum diameter, tumor volume, invasiveness, as well as T2-weighted signal features did not significantly differ between patients achieving biochemical control (tight and less stringent definition) and uncontrolled subjects.

**Figure 3:**
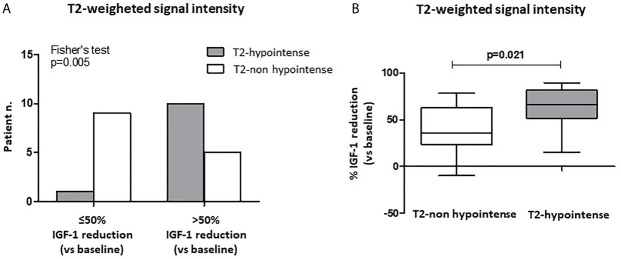
Biochemical response after first-line SRL treatment (6 months) with respect to T2-weighted signal intensity at MRI of the sella turcica. A higher percentage of patients with T2-weighted hypointense tumors at MRI achieved a >50% IGF-1 reduction after 6-month SRL treatment, compared to those harboring a non-hypointense lesion **(A)**. Patients with a T2-hypointense tumor had a higher % IGF-1 reduction compared to the subjects with a non-hypointense lesion **(B)**. SRL, somatostatin receptor ligand.

After 6-month treatment with fg-SRLs, a >50% IGF-1 reduction was reached in a higher percentage of patients with T2-weighted hypointense tumors (10/11, 90%), compared to those harboring a non-hypointense lesion (5/14, 36%) (p=0.005) (**Figure 3A**). Furthermore, patients with a T2-hypointense tumor experienced a higher % IGF-1 reduction compared to the subjects with a non-hypointense lesion [65.8% (IQR 51.5-81.4) vs. 35.5% (IQR 23.4-62.9); p=0.021] (**Figure 3B**).

#### Diagnostic and Dynamic Tests

**Figure 4:**
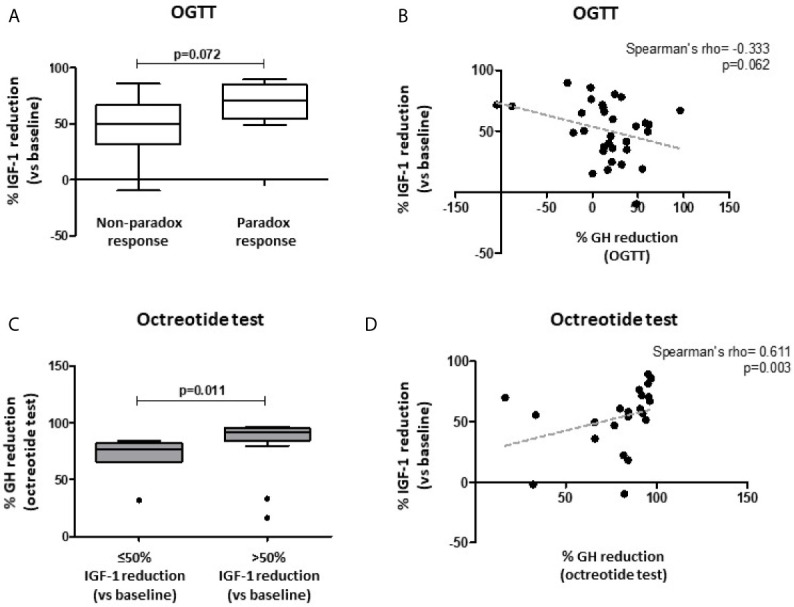
Biochemical response after first-line SRL treatment (6 months) with respect to patients’ response to both oral glucose tolerance test (OGTT) and acute octreotide test. Patients with a paradoxical increase of GH levels during OGTT (≥ 20% GH increase vs. baseline) showed a trend towards a higher % IGF-1 reduction after fg-SRL treatment, compared with the non-paradoxical group **(A)**. A trend for an inverse correlation between the % GH reduction after OGTT (nadir vs. baseline) and the % IGF-1 reduction after 6-month SRL treatment was observed **(B)**. Patients with higher % GH reduction after acute octreotide test (nadir vs. baseline) were more likely to reach a >50% IGF-1 reduction, compared to those with a lower response rate **(C)**. The % GH reduction after acute octreotide test was directly correlated with the % IGF-1 reduction after 6-month SRL therapy **(D)**. OGTT, oral glucose tolerance test; fg-SRLs, first-generation somatostatin receptor ligands.

Complete data of OGTT performed at diagnosis were available in 32 patients. We observed a trend for an inverse correlation between the % GH reduction after OGTT (nadir vs. baseline) and the % IGF-1 reduction after 6-month SRL treatment (r=-0.333, p=0.062) (**Figure 4B**). Furthermore, patients with a paradoxical increase of GH levels during the OGTT, showed a higher % IGF-1 reduction, although not statistically significant, compared with the non-paradoxical group [71.2% (IQR 54.3-85.2) vs. 49.9% (IQR 31.4-66.8); p=0.072] (**Figure 4A**).

Among patients which underwent an acute octreotide tolerance test, the % GH reduction (nadir vs. baseline) was significantly higher in those patients achieving tight biochemical control (IGF-1 ≤1 xULN) compared to the uncontrolled ones [95.3% (IQR 91.1-95.9) vs. 81.6% (IQR 65.9-84.3); p=0.003]. Similarly, patients reaching biochemical control (less stringent definition, IGF-1 ≤1.2 xULN) showed a higher % GH reduction during the acute test, compared to those patients with an active disease [93.6% (IQR 82.6-95.6) vs. 82.9% (IQR 57.7-91.2); p=0.004]. Moreover, patients with higher % GH reduction were more likely to reach a >50% IGF-1 reduction, compared to those with a lower response rate [91.8% (IQR 84.2-95.3) vs. 76.6% (IQR 65.8-82.1); p=0.011] (**Figure 4C**).

In line with these observations, the % GH reduction after acute octreotide test was directly correlated with the % IGF-1 reduction after 6-month SRL therapy (Spearman’s rho=0.611, p=0.003) (**Figure 4D**).

### Univariable and Multiple Regression Models

#### Logistic Regression Analyses

The results of univariable logistic regression analyses, evaluating the association between the candidate predictors of biochemical response (classified as tight biochemical control, biochemical control, and >50% IGF-1 reduction), are shown in **Table 2**. Candidate predictors were selected performing summary statistics, and then between-group comparisons, cross-tables and correlation tests (see *Statistical Analysis* section).

**Table 2:** Univariable and multivariable logistic regression analysis to predict biochemical control.

Univariable logistic regression			
Dependent Variables	Independent variables	OR (95%CI)	p value
**Tight biochemical control** (IGF-1 ≤1 xULN)	% GH reduction after acute octreotide test	1.048 (0.980-1.120)	0.175
**Biochemical control** (IGF-1 ≤1.2 xULN)	% GH reduction after acute octreotide test	1.047 (0.983-1.115)	0.152
**IGF-1 reduction >50%**	Age ≤40 yrs	0.04 (0.00 – 0.93)	**0.045**
	BMI	0.866 (0.758-0.989)	**0.034**
	IGF-1 xULN	2.304 (1.254-4.234)	**0.007**
	T2-hypointense signal	18 (1.754-184.679)	**0.015**
	% GH reduction after acute octreotide test	1.024 (0.984-1.066)	0.237
**Multivariable logistic regression**			
**Dependent Variables**	**Independent variables**	**Adjusted OR (95%CI)**	**p value**
**IGF-1 reduction >50%**	Age ≤40 yrs	0.04 (0.01 – 0.95)	**0.047**
	IGF-1 xULN	5.20 (0.94-28.61)	**0.058**
	T2-hypointense signal	29.45 (0.63-1381.50)	**0.085**

OR, odds ratio; 95%CI, 95% confidence interval; BMI, body mass index; IGF-1, insulin-like growth factor 1; ULN, upper limit of normality range; yrs, years. Bold values are statistically significant (p-value ≤ 0.05).

A better response to the acute octreotide tolerance test (namely, higher % GH reduction) emerged as a candidate predictor for both tight (IGF-1 ≤1 xULN) and less stringent (IGF-1 ≤1.2 xULN) biochemical control after 6-month SRL treatment. However, at univariate logistic regression analysis this variable failed to reach statistically significant odds [OR 1.047 (95%CI 0.983-1.115) and OR 1.048 (95%CI 0.980-1.120), respectively, **Table 2**].

On the other hand, when evaluating the predictors for a >50% IGF-1 reduction, more candidate variables were identified (e.g. dichotomized age, BMI, baseline IGF-1 xULN values, T2-hypointense signal, % GH reduction after acute octreotide test). Younger age [OR 0.04 (95%CI 0.00-0.93)] and higher BMI [OR 0.866 (95%CI 0.758-0.989)] were associated with a lower chance of achieving >50% IGF-1 reduction after 6-month SRL treatment (**Table 2**). On the contrary, higher IGF-1 xULN values at diagnosis [OR 2.304 (95%CI 1.254-4.234)] and a T2-weighted hypointense signal of the pituitary tumor [OR 18 (95%CI 1.754-184.679] were associated with a significantly higher likelihood of achieving a >50% IGF-1 reduction after SRL therapy (**Table 2**).

Statistically significant predictors at univariable analyses were included in the multivariable model. Both feature selection methods (see *Statistical Analysis* paragraph) identified dichotomized age, IGF1 xULN at diagnosis, and T2-hypointense signal of the tumor to be retained by our multivariable logistic regression model (p<0.1, **Table 2**), while BMI was excluded. Overall, age >40 years, IGF-1 xULN at diagnosis and T2-hypointense pituitary tumor had an excellent discriminative ability to predict a >50% IGF-1 reduction after 6-month SRL treatment (AUC 0.98, 95%CI 0.93-1.0) (**Supplementary Figure 1**).

#### Linear Regression Analyses

Univariable regression analysis to predict the % IGF-1 reduction after 6-month fg-SRL treatment found a negative association with younger age (≤ 40 years; β -0.533, p<0.0001), while a positive association was observed with both IGF-1 xULN levels at diagnosis (β 0.330, p= 0.018) and the presence of a T2-weighted hypointense signal of the pituitary lesion (β 0.466, p=0.019) (**Table 3**).

**Table 3:** Univariable and multivariable linear regression analysis for the predictors of relative IGF-1 reduction (percent reduction after 6-month SRL treatment).

Univariable linear regression analysis					
Dependent Variable	Independent Variables (IVs)	Adjusted R^2^	B	β	p value
**Relative IGF-1 reduction (%)**	Age ≤40 yrs	0.269	-40.08	-0.533	**<0.0001**
	IGF-1 xULN	0.091	6.985	0.330	**0.018**
	T2-hypointense signal	0.184	24.71	0.466	**0.019**
	% GH reduction after OGTT	0.069	-0.187	-0.315	0.079
	OGTT paradox response	0.071	22.158	0.317	0.077
**Multivariable linear regression analysis**					
**Dependen Variable**	**Independent Variables (IVs)**	**Adjusted R^2^**	**B**	**β**	**p value**
**Relative IGF-1 reduction (%)**	All IVs	0.695	–	–	**<0.0001**
	Age ≤40 yrs	–	-46.927	-0.654	**<0.0001**
	IGF-1 xULN	–	7.278	0.303	**0.019**
	T2-hypointense signal	–	13.735	0.259	**0.043**

yrs, years; BMI, body mass index; IGF-1, insulin-like growth factor 1; ULN, upper limit of normality range; OGTT, oral glucose tolerance test. Bold values are statistically significant (p-value ≤ 0.05).

All these variables were still significant predictors at multivariable analysis, showing an overall adjusted R^2^ of 0.695 (**Table 3**).

Of note, the results of multivariable linear regression analysis were confirmed when adding drug dose as an additional independent variable affecting SRL response (**Supplementary Table 1**).

## Discussion

In the present study we identified relevant clinical, biochemical, and radiological predictors of response to first-line treatment (6 months) with fg-SRLs in a cohort of acromegaly patients referred to our Institution. To our knowledge, this is the first study evaluating in a real-life setting a large number of different potential predictors possibly associated with the response to SRL treatment in naïve acromegaly patients (>20 variables screened).

Differently from most studies reported in the literature, we investigated biochemical response evaluating IGF-1 levels both as categorical (control, tight control, >50% reduction) and continuous (% IGF-1 reduction) variables. Of note, IGF-1 (and GH) measurements reported in the present study were all measured by using the same assay, in the same laboratory.

We focused on IGF-1 since most recent Consensus Statements on acromegaly suggest it as the main target hormone to monitor disease activity (10), being more constant than GH in a single measurement, without showing significant circadian variations (46–48).

Using a rigorous approach, the candidate predictors were selected based on summary statistics, and then using between-group comparisons, cross-tables and correlation tests. Afterwards, the selected variables were included in the different prediction models, performing univariable and multivariable logistic regression and linear regression analyses.

In our cohort, we failed to identify robust predictors of both tight (IGF-1 ≤1 xULN) and less stringent (IGF-1 ≤1.2 xULN) biochemical control. In this setting, % GH reduction after acute octreotide test (nadir vs. baseline) emerged as the best potential predictor of biochemical response, being significantly higher in patients achieving normal age-adjusted IGF-1 levels, compared to those with active disease (Mann-Whitney test). However, at univariate logistic regression this variable did not show any significant predictive value.

On the other hand, when considering a >50% IGF-1 reduction as treatment outcome, we found that younger age and higher BMI were associated with a lower response to fg-SRL treatment, while higher IGF-1 xULN at diagnosis and the presence of a T2-hypointense pituitary tumor were associated with a better response to treatment (all statistically significant at univariable analysis).

These findings are in line with previous data from the literature, obtained from different reports although showing a wide heterogeneity in study design and sample size (21–24, 27, 28, 49).

When performing multivariable logistic regression analysis, we dealt with some statistical issues due to the presence of a dichotomous variable exhibiting a “perfect separation” with the outcome >50% IGF-1 reduction (no young patients showed good response to SRLs), as well as with a limited number of complete observations in our model (T2-weighted signal intensity was available in 25 subjects). Anyhow, performing adequate corrections to the statistical model, we demonstrated that younger age was still significantly associated with a lower chance to achieve a satisfactory response to first-line SRL treatment, while higher IGF-1 xULN at diagnosis and T2-hypointense signal of the tumor both maintained a trend towards a higher chance to have a better response. To our opinion, this is of particular interest, since we are facing with real life data, with all their intrinsic limitations.

Again, evaluating the predictors of relative IGF-1 reduction after treatment (% IGF-1 reduction after 6-month therapy), we confirmed that older age, higher baseline IGF-1 xULN and T2-hypointense signal of the tumor were the best predictors of biochemical response, both at univariable and multivariable linear regression analyses.

In this context, we have to highlight that the association between higher IGF-1 levels at baseline and a better response to SRL treatment is in line with the recent findings of Coopmans and colleagues, which investigated the predictors of biochemical response in a large number of subjects from two different cohorts (>600 patients) (21).

These results are interesting from a clinical perspective, since they point out that patients with higher baseline IGF-1, although not reaching the threshold set to define biochemical control, show a greater relative reduction of IGF-1 levels, and therefore could benefit of SRL treatment in the context of a multimodal therapy.

Furthermore, this finding is not at odds with previous studies describing an association between lower IGF-1 levels at baseline and a higher percentage of biochemical control after SRL treatment (21–24, 50). Indeed, although showing a lower relative reduction of hormone secretion, patients with lower baseline IGF-1 levels are (obviously) more likely to achieve the threshold set for biochemical control.

Based on previous data from the literature, the link between IGF-1 levels at diagnosis and the MRI features of the pituitary tumor could, at least partially, explain our findings. Indeed, it has been largely described that T2-hypointense tumors show higher GH and IGF-1 levels at baseline compared to non-hypointense lesions (27, 28, 49, 51, 52). Furthermore, some authors have already suggested an association between T2-hypointense tumors and a densely granulated pattern of the lesions, while T2-hyperintense tumors seem to be more frequently sparsely granulated (51–53).

In this light, densely granulated tumors usually show a better response to SRL treatment compared to the sparsely granulated ones, possibly due to a relatively higher expression of SST_2_ on tumor cell membrane, as well as a reduced E-cadherin immunoreactivity (54, 55). This could explain the better response to SRL therapy, in terms of relative IGF-1 reduction, observed for T2-hypointese tumors, which in turn are associated with higher baseline IGF-1 levels.

Besides clinical, biochemical, and radiological characteristics, we have investigated the potential role of dynamic tests (OGTT at diagnosis and acute octreotide test) as predictors to first-line SRL treatment. In line with recent data from other authors, we observed that a lower % of GH suppression after OGTT or the presence of a paradoxical GH increase (≥20% vs. baseline) are associated (with a trend towards statistical significance) with a better response to fg-SRL therapy (45). Of note, Scaroni and colleagues observed that patients showing a paradox response to OGTT also had higher baseline IGF-1 xULN values and were older compared to those subjects with a non-paradoxical response (45).

On the other hand, the results of the acute octreotide tests are difficult to interpret. Indeed, despite the meaningful differences identified when performing median-comparison tests or correlation analyses, linear and logistic regression modeling revealed no significant association with the selected outcomes. This issue is in line with the controversial results reported in the literature about the potential clinical impact of this test, as a reliable predictor of fg-SRL treatment response.

Our study presents some limitations, such as the retrospective study design. Indeed, due to the retrospective data collection, not all parameters were available for all patients (e.g. radiological characteristics and results of the acute octreotide test). However, since our findings are largely in line with previous data from the literature, the relatively low number of patients evaluated for some specific variables could have not significantly affected our analyses.

On the other hand, a strength of our study is the careful evaluation of a large number of potential predictors (clinical, biochemical and radiological parameters, as well as dynamic tests) of biochemical response to fg-SRLs as first-line treatment in acromegaly, performed at a single referral center for pituitary diseases. Furthermore, the same well-validated assay was used to assess all IGF-1 measurements included in the study. This was made possible since in our Institution we had a high percentage of patients treated with first-line SRL therapy.

In conclusion, we found that younger patients (≤40 years-old) are less likely to achieve a relevant IGF-1 reduction after first-line SRL treatment, while higher IGF-1 values at diagnosis and a T2-hypointense lesion at MRI are robust predictors of biochemical response (namely, IGF-1 reduction).

Taken together, these three parameters (dichotomized age, IGF-1 levels, and tumor T2-weighted signal) can provide a satisfactory discriminative ability to predict a >50% IGF-1 reduction after 6-month fg-SRL treatment.

Since this information can be available in all referral centers for pituitary diseases, such evaluations should be considered in the light of an individualized treatment plan for acromegaly patients, in order to minimize the currently predominant “trial-and error” approach.

## Data Availability Statement

The raw data supporting the conclusions of this article will be made available by the authors, without undue reservation.

## Ethics Statement

Ethical review and approval was not required for the study on human participants in accordance with the local legislation and institutional requirements. The patients/participants provided their written informed consent to participate in this study.

## Author Contributions

FG: Conception and Design, Acquisition of Data, Analysis and Interpretation of Data, Statistical analysis, Drafting the Article, Critically Revising the Article, Reviewed submitted version of manuscript, Study supervision. FN: Conception and Design, Acquisition of Data, Analysis and Interpretation of Data, Statistical analysis, Drafting the Article, Critically Revising the Article, Reviewed submitted version of manuscript. GC: Acquisition of Data, Analysis and Interpretation of Data, Reviewed submitted version of manuscript; LC: Acquisition of Data, Analysis and Interpretation of Data, Study supervision. KK: Acquisition of Data, Analysis and Interpretation of Data, Reviewed submitted version of manuscript. CC: Acquisition of Data, Analysis and Interpretation of Data, Reviewed submitted version of manuscript. FC: Acquisition of Data, Analysis and Interpretation of Data, Reviewed submitted version of manuscript. GaZ: Analysis and Interpretation of Data, Statistical analysis, Critically Revising the Article. AP: Conception and Design, Analysis of Data, Critically Revising the Article, Reviewed submitted version of manuscript. DR: Conception and Design, Analysis of data, Reviewed submitted version of manuscript, Study supervision. GiZ: Analysis and Interpretation of data, Critically Revising the Article, Reviewed submitted version of manuscript. DF: Conception and Design, Analysis and Interpretation of Data, Statistical analysis, Critically Revising the Article, Reviewed submitted version of manuscript, Administrative/technical/material support, Study supervision. All authors contributed to the article and approved the submitted version.

## Funding

This research did not receive any specific grant from any funding agency in the public, commercial or not-for-profit sector.

## Conflict of Interest

DF received grants and fees for lectures and participation to advisory boards for Novartis, Ipsen and Pfizer. F.G. received fees for lectures and/or participation to advisory boards for Novartis, AMCo, and IONIS Pharmaceuticals.

The remaining authors declare that the research was conducted in the absence of any commercial or financial relationships that could be construed as a potential conflict of interest.
