# Supplementary material for: Clinical and Radiological Predictors of Biochemical Response to First-Line Treatment With Somatostatin Receptor Ligands in Acromegaly: A Real-Life Perspective
Source: Front Endocrinol (Lausanne). 2021 May 7;12:677919. doi: 10.3389/fendo.2021.677919 (PMC8139627; doi:10.3389/fendo.2021.677919)
Supplement: Supplementary file 1 [file Presentation_1.pptx]

## Slide 1
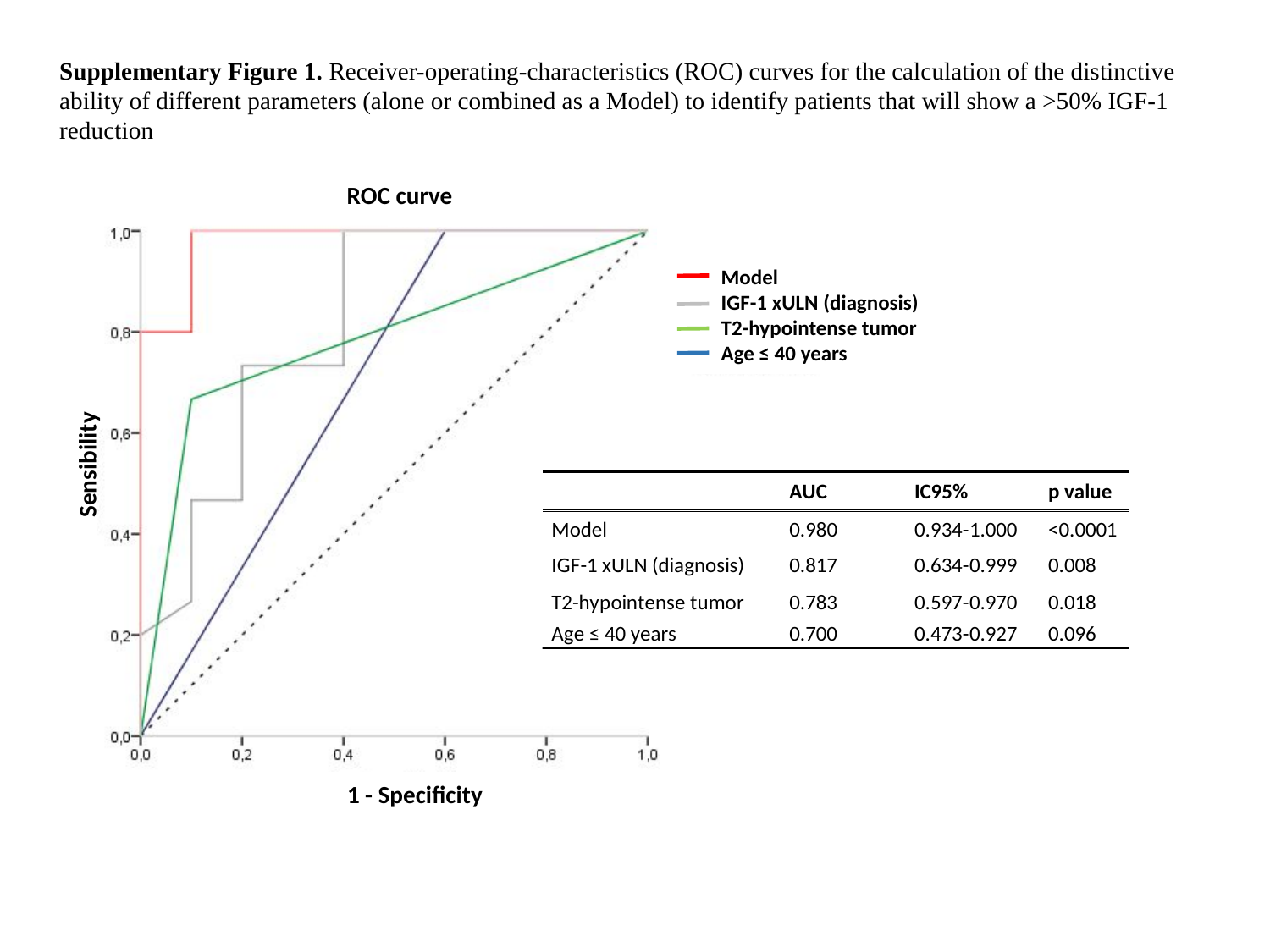

Supplementary Figure 1. Receiver-operating-characteristics (ROC) curves for the calculation of the distinctive ability of different parameters (alone or combined as a Model) to identify patients that will show a >50% IGF-1 reduction
ROC curve
Model
IGF-1 xULN (diagnosis)
T2-hypointense tumor
Age ≤ 40 years
Sensibility
1 - Specificity
