# Supplementary material for: Clinical and Radiological Predictors of Biochemical Response to First-Line Treatment With Somatostatin Receptor Ligands in Acromegaly: A Real-Life Perspective
Source: Front Endocrinol (Lausanne). 2021 May 7;12:677919. doi: 10.3389/fendo.2021.677919 (PMC8139627; doi:10.3389/fendo.2021.677919)
Supplement: Supplementary file 2 [file Table_1.docx]

| **Multivariable linear regression analysis** | | | | | |
| --- | --- | --- | --- | --- | --- |
| Dependent  Variable | Independent  Variables (IVs) | Adjusted R^2^ | B | β | p value |
| **Relative IGF-1 reduction (%)** | All IVs | 0.684 | - | - | **<0.0001** |
|  | Age ≤40 yrs | - | -45.923 | -0.640 | **<0.0001** |
|  | IGF-1 xULN | - | 6.909 | 0.288 | **0.033** |
|  | T2-hypointense  signal | - | 13.611 | 0.257 | **0.049** |
|  | Drug dose | - | -3.418 | -0.058 | 0.645 |

**Supplementary Table 1.** Multivariable linear regression analysis for the predictors of relative IGF-1 reduction (% reduction after 6 month SRL treatment) adjusted for SRL dose.
